# Identifying Big Five personality traits based on facial behavior analysis

**DOI:** 10.3389/fpubh.2022.1001828

**Published:** 2022-09-09

**Authors:** Lei Cai, Xiaoqian Liu

**Affiliations:** ^1^Institute of Psychology, Chinese Academy of Sciences, Beijing, China; ^2^Department of Psychology, University of Chinese Academy of Sciences, Beijing, China

**Keywords:** facial key point, personality trait identification, Big Five, machine learning, noninvasive identification

## Abstract

The personality assessment is in high demand in various fields and is becoming increasingly more important in practice. In recent years, with the rapid development of machine learning technology, the integration research of machine learning and psychology has become a new trend. In addition, the technology of automatic personality identification based on facial analysis has become the most advanced research direction in large-scale personality identification technology. This study proposes a method to automatically identify the Big Five personality traits by analyzing the facial movement in ordinary videos. In this study, we collected a total of 82 sample data. First, through the correlation analysis between facial features and personality scores, we found that the points from the right jawline to the chin contour showed a significant negative correlation with agreeableness. Simultaneously, we found that the movements of the left cheek's outer contour points in the high openness group were significantly higher than those in the low openness group. This study used a variety of machine learning algorithms to build the identification model on 70 key points of the face. Among them, the CatBoost regression algorithm has the best performance in the five dimensions, and the correlation coefficients between the model prediction results and the scale evaluation results are about medium correlation (0.37–0.42). Simultaneously, we executed the Split-Half reliability test, and the results showed that the reliability of the experimental method reached a high-reliability standard (0.75–0.96). The experimental results further verify the feasibility and effectiveness of the automatic assessment method of Big Five personality traits based on individual facial video analysis.

## Introduction

Personality refers to the internal tendency and psychological characteristics of an individual's behavior in social adaptation to people, things, and themselves. Personality affects various aspects such as individual consumption habits, performance ability, interpersonal communication, mental health, and even political stance. Personality assessment has a variety of applications in different fields. Researchers have undertaken a substantial amount of studies on personality, and these studies have covered a variety of areas, such as the relationship with emotional expression identification (1); personality, affective and cultural aspects (2); healthy behavioral patterns prediction of depression and anxiety (3); prediction of leader performance (4); prediction of compulsive shopping (5); sales prediction of performance (6); achievement motivation (7); and the prediction of political attitudes (8). In addition, it has also been used in a variety of industries such as tourism (9), medicine (10), education (11), and finance (12). However, those studies on personality measurement were primarily based on scales [such as the Minnesota Multiphasic Personality Inventory (MMPI), the Eysenck Personality Questionnaire (EPQ), the Cattell's 16 Personality Factors Test (16PF), the Edwards Personality Preference Schedule (EPPS), the Five-Factor Personality Inventory (FFPI), and the Big Five Inventory (BFI), et al.]. Although traditional personality scales have the characteristics of high accuracy, scale measurement costs more resources (people and time) and is hard to implement on a large scale. Therefore, this study attempts to find a more efficient, effective, and large-scale test of personality identification method than traditional personality scales.

This paper mentioned that personality traits have been applied to various industries and can increase productivity. Therefore, more enterprises, especially Internet enterprises, have realized that identifying users' personality traits can help improve and optimize enterprise services. However, due to the need for the active participation of users, the feasibility of obtaining the user's personality through self-report is very low on scale measurement. Therefore, in order to solve the problem that users are unwilling to self-report, we propose a non-invasive method for predicting personality scores from videos. With the widely used social media (i.e., Tik Tok), the network contains a large amount of user-shared video data. In those videos, The user's facial movement contains individual personality information. Analyzing this information can identify personal personality traits. On the internet, adolescents are dominant, and their personality and mental health are closely related. Enterprises may provide better mental health services based on non-invasive personality identification. This non-intrusive user personality identification method based on Internet user videos has a significant application value.

Automatic identification of personality traits through machine learning based on various media is a popular research direction recently, such as text (13), images (14–16), morphological analysis (17), and social media (18). This study proposes a novel method to identify personality based on video streams and analyzes the changes in facial expressions when users speak in natural scenes. In this way, speaking under normal circumstances, the expression of users' emotions is more natural and ecological, and there is a close relationship between facial emotional expression and personality traits (1). This method is more realistic than text identification because the latter has been affected by the user's cultural level and expressive ability. Compared with image identification, It's more continuous. And it's simpler than morphological analysis, which requires 3D analysis tools and methods. In addition, Identifying personality *via* social media is primarily based on text, images, and users' personal information (such as career, age, hobbies, et al.). It has the disadvantages of text and image identification and requires users to report their private information voluntarily. Therefore, identifying personality by analyzing facial videos is more accurate and effective than the above methods. Previous research has used CNN (19–21) on facial videos to identify personality, and it worked effectively. In this study, compared with the unsupervised model of CNN, we used several machine learning models of supervised learning. This method helps further analyze which areas of the face are closely related to personality. In addition, this study conducts reliability and validity tests on this method, which can better prove the validity and reliability of this method.

In this study, we chose the BFI as a supplementary scale for personality identification. The BFI contains five dimensions, Agreeableness (A), Conscientiousness (C), Extraversion (E), Neuroticism (N), and Openness (O). The BFI is the most frequently used scale in various research, and the accuracy rate is very high. This study uses the BFI-44 scale, which ensures accuracy and improves operability. The BFI-44 contains 44 questions that examine five dimensions. Unlike other scales with nearly 100 questions, 44 questions are not easy to cause the subjects to be overtired when doing the questions and avoid unpredictable random errors. In addition, unlike scales with too few items, it can also accurately test the personality characteristics of the subjects. The subjects' BFI-44 scores were used for machine learning training.

By obtaining a small number of subject BFI-44 scale scores and correlating them with the subjects' video data, we use five commonly used machine learning supervised models for training. This is undertaken to predict other subjects' personality scores through the machine learning model based on the video data of experimental subjects. The advantage of our method for automatic personality identification is that it can be identified on a large scale and is not easily affected by the subject subjective impressions and learning effects.

## Materials and methods

### Subject recruitment

The subjects were recruited by publishing announcements. The recruited subjects were required to be above 18 years old, disease-free (free from any mental illness identified by a psychiatrist, no disease affecting the face and facial expressions, and in good health), and with regular language expression ability. Through sample screening, data denoising, and other operations, 82 test samples were obtained from the total 88 samples for this study. This included 27 females and 55 males. The average age was 22.41 years old, and the age variance was 17.58 years old. Note that all the subjects we recruited are Chinese. The demographic information of the subjects is provided in Table 1.

**Table 1:** Demographic information of subjects.

**Demographic information**	***n***	***%***
**Gender**		
Female	55	67.07%
Male	27	32.93%
**Marital status**		
Single	48	58.54%
Partnered	30	36.59%
Married	2	2.44%
Divorced	2	2.44%
**Educational background**		
Graduated from senior high school or technical secondary school	2	2.44%
College student	33	40.24%
Graduate / Vocational college graduation	21	25.61%
Graduate student	20	24.39%
Master/PHD	6	7.32%

Mean = 22.41, Variance = 17.58.

### Data collection

First, we introduced the purpose and steps of the experiment to the subjects according to the instruction language. Subsequently, we asked the subjects to sign an informed consent form and registered the subjects' personal information, and presented them with a speech outline as follows:

(1) Please introduce yourself and your hometown in detail.
(2) Please introduce your major and your research work during your studies in detail, or Introduce your current industry and specific job content.
(3) Please tell us about your future plans and what kind of work you would like to do.

**Figure 1:**
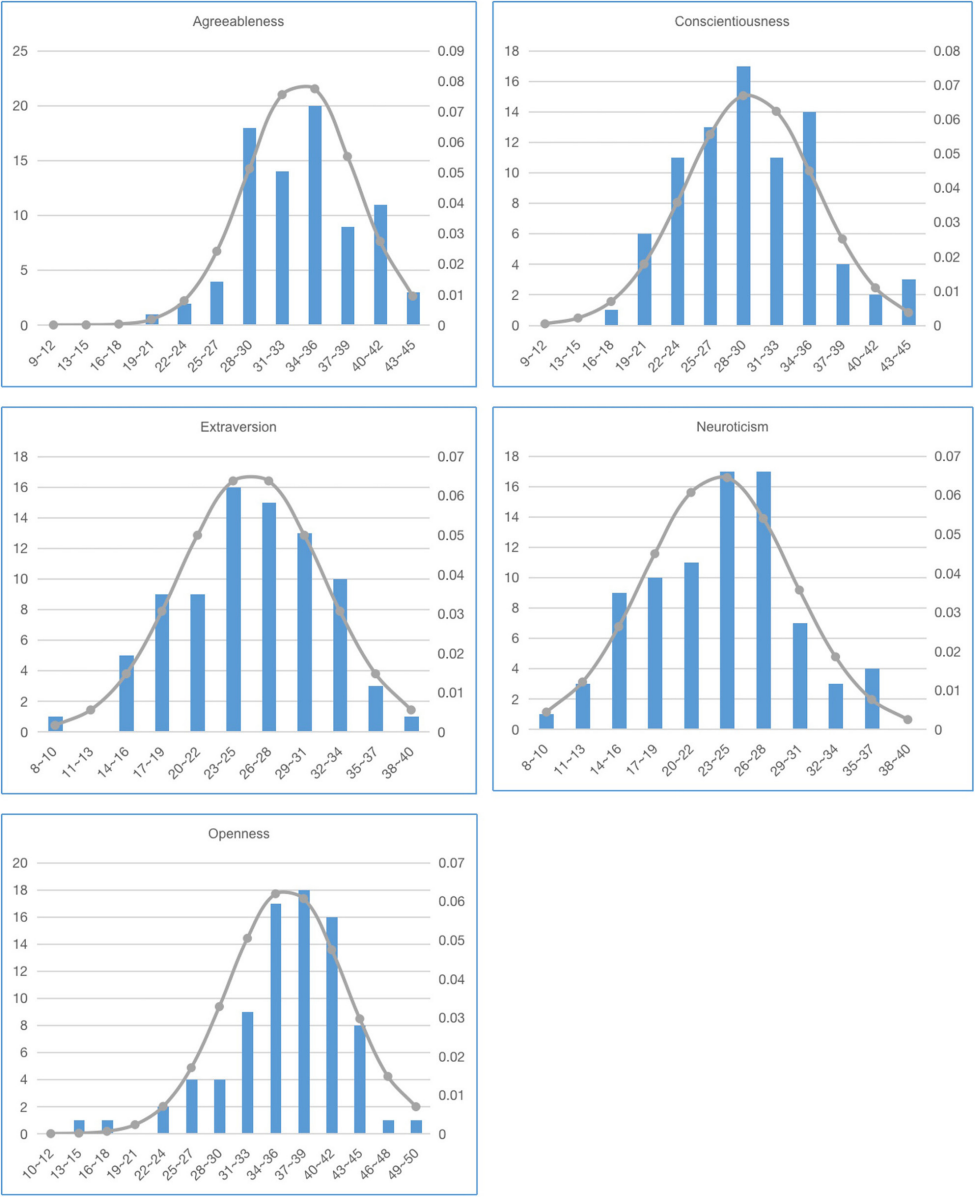
The score distribution of BFI-44 on five dimensions.

Second, the subjects had 5 min to prepare. Subsequently, the subjects completed a speech of at least one and a half minutes, and we used a camera to record the subjects' facial movements during their speech. Subsequently, all subjects need to fill in the BFI-44. The score distribution of BFI-44 from them are shown in Figure 1.

### Data processing

**Figure 2:**
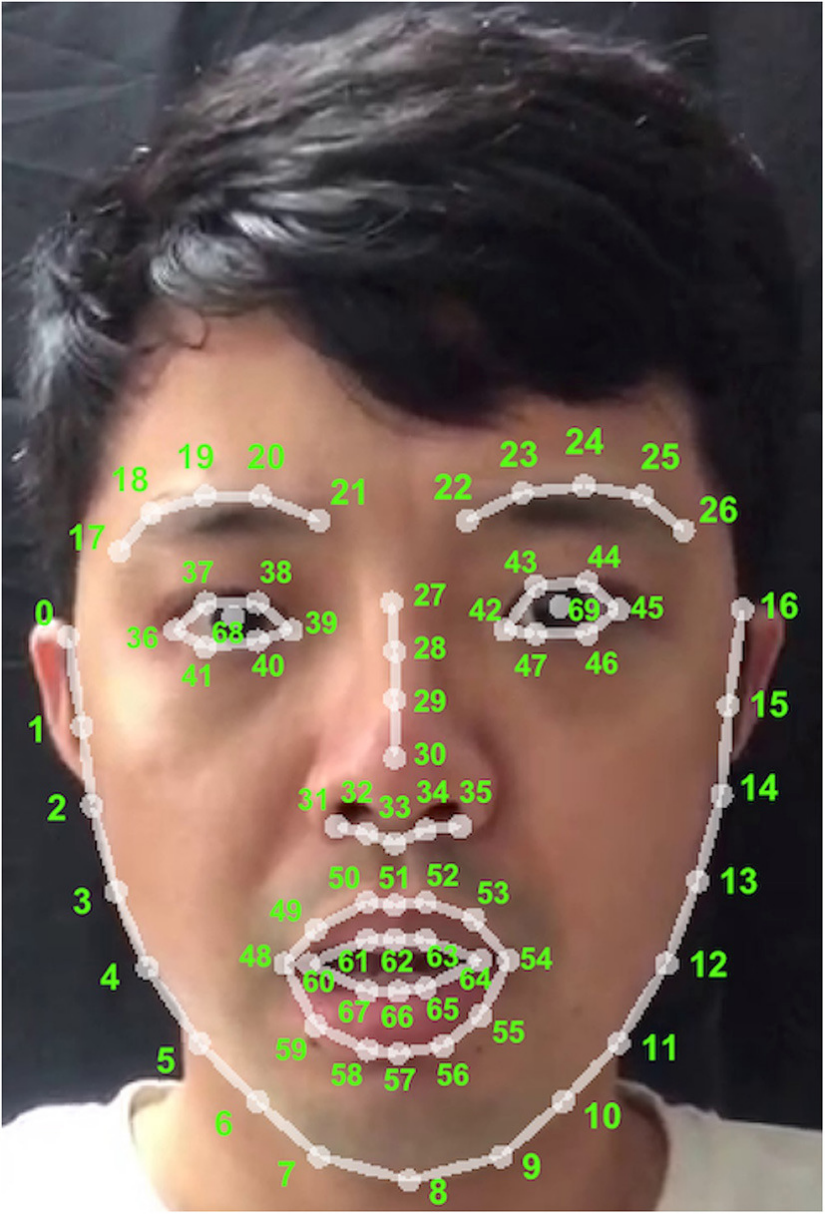
The 70 key points on face.

In this study, we used the Openpose (22) open source code package to process the video and obtained the two-dimensional coordinates (*x, y*) of the 70 key points of the face (Figure 2) of the subjects in each frame of the video. The “*x*” represents the pixel distance between the key point and the zero coordinate (0, 0) in the upper left corner of the each frame graph of the user's video in the horizontal direction, and the *y* point represents the pixel distance between the key point and the zero coordinate in the vertical direction. The frame rate of the video is 25 frames per second. Means the camera can record 25 frames of coordinate tracks of each key point in 1 s.

Considering that the subjects had a transition period to enter the experimental state at the beginning of the recording, the facial changes during this transition period may have produced random errors. Consequently, to reduce or avoid these errors, we removed the first 4 s (100 frames) of the video.

### Data smoothing

During the video recording process, due to the micro-motion reaction of the face, the slight vibration of the environment, and the change of light, the test subjects were affected, causing random errors. Therefore, we used mean smoothing to reduce random errors. The average smoothing process takes the current frame of the face key point P as the center and uses a certain number of W image frames adjacent to the same P point as a window (*W* = 3 in this experiment). Subsequently, it calculates the average value of all P points in a window. Then the average value of the *x*-axis or *y*-axis data is used as the new value of point P, and it is calculated every frame by sliding the window backward.

### Frame difference processing

Due to the height difference of each subject, the starting position of the subject's face was also different when they were recorded. We used the frame difference method to subtract the coordinates of the previous frame of the point P from the coordinates of the current frame of the point P. Subsequently, we took the absolute value and finally found the frame difference coordinates of the point P on the *x*-axis and the *y*-axis respectively. The data processed by the frame difference can avoid systematic errors caused by the difference in the height of the subjects, and simultaneously, we focused on the changing trends of the subjects' faces on a time axis.

### Select foundation data

We selected the data of *N* frames (*N* can be 500, 750, or 1,000 frames; *N* = 750 used in this experiment) as the foundation data. It's 70 key points on the face. Each of them is represented by *p*^*i*^, where the letter i represents the serial number of key points from 0 to 69, and the numbers of all key points are shown in Figure 2. Each foundation data contains 750 frames. Each frame includes the *x* and *y* coordinates of the key points. Represent as *p*^*i*^ ($x_{n}^{i}$, $y_{n}^{i}$), where the letter i is the serial number of the key point and the letter *n* is the frame serial number.

### Remove noise data

When subjects are recording, irrelevant body movements affect the research data, such as turning the entire head and nodding the head up and down. This study primarily analyzed the relationship between facial movements and the Big Five personality traits, and these irrelevant limb movements can create data noise. In machine learning, the existence of noise seriously affects the training effect of the model. It results in a decrease in the prediction effect of the model. Therefore, it is necessary to process the noise data. We calculated the mean of the feature dimensions obtained by each sample, identified the data that exceeded the mean value by three standard deviations as noise data, and deleted the noise data. There are 88 samples in total. Six noise samples were removed, and 82 valid sample data were retained final.

### Correlation analysis between facial key point movement and personality score

To explore the relationship between facial activity and personality. We performed a correlation analysis between facial movement and the scores of the BFI-44 and a high-low group *T*-test. It was used to support the technical feasibility of personality identification based on facial video analysis. Consequently, we calculated the mean and variance of the displacement change of each key point in the *X*-axis and *Y*-axis based on 750 frames of data. The mean and variance of the ith point on the *X*-axis and *Y*-axis are represented by P^*i*^_X_MEAN, P^*i*^_X_VAR, P^*i*^_Y_MEAN, P^*i*^_Y_VAR. Each subject has 280(70^*^2^*^2) features, consisting of 70 key points, two dimensional coordinates, and two types of values (Mean and Variance). Therefore 82 subjects can obtain a two-dimensional array of 82^*^280 data. The mean feature can reflect the average magnitude of the subject's facial movement in each frame, and the variance feature can reflect the intensity of their facial movement. In the correlation analysis, after calculating the Pearson correlation coefficient *R*-value and *p*-value with the mean and variance characteristics and the scores of the five dimensions of the BFI-44 of the 82 samples, we found that facial movement was significant in the Agreeableness dimension. However, not a case (*p* < 0.05) was found in the other four dimensions (Table 2). The significant performance of Agreeableness is predominantly concentrated in the *X*-axis variance of points four to 10 and the *Y*-axis variance of point nine. In addition, we found a negative correlation between facial motion and Agreeableness. According to the 4–10 point distribution map, these points are primarily distributed from the right jawline to the chin contour. Therefore, Agreeableness is significantly correlated with the movement of the right jawline contour of the face to the chin contour.

**Table 2:** Correlation analysis of facial key point movement and personality score.

**Feature of face keypoint**	**Agreeableness**
P4_X_VAR	−0.231^*^
P5_X_VAR	−0.219^*^
P6_X_VAR	−0.222^*^
P7_X_VAR	−0.246^*^
P8_X_VAR	−0.246^*^
P9_X_VAR	−0.233^*^
P9_Y_VAR	−0.226^*^
P10_X_VAR	−0.221^*^

* p < 0.05.

In the *T*-test for high and low grouping, the data processing is similar to that of correlation analysis, except that we divided the original 82 samples into two samples based on the five-dimensional scores of the BFI-44. In addition, Each dimensional of BFI-44 has two samples(A and B), A is the top 27% of the sample with the highest score and B is the top 27% of the sample with the lowest score. *T*-test was performed on samples A and B of each dimensions of BFI-44. The results indicated that only the Openness dimension showed significant differences (Table 3). Furthermore, the difference highlighted a positive correlation. In addition, we found that Openness is significantly related to points 11–15 on the face, and 11–15 are mainly distributed on the left cheek's outer contour. Therefore, Openness is significantly related to the movement of the left cheek's outer contour.

**Table 3:** High-low grouping *T*-test.

**Feature of face key point**	**Openness**					
	**High grouping**		**Low grouping**		***T*-value**	***p*-Value**
	**Mean**	**Variance**	**Mean**	**Variance**		
P11_Y_VAR	0.149	0.007	0.103	0.003	2.126	0.039
P12_Y_MEAN	0.4	0.008	0.334	0.007	2.503	0.016
P13_Y_MEAN	0.384	0.007	0.317	0.008	2.496	0.017
P13_Y_VAR	0.145	0.006	0.096	0.003	2.297	0.027
P14_Y_MEAN	0.392	0.008	0.328	0.008	2.225	0.031
P15_Y_MEAN	0.395	0.006	0.331	0.01	2.319	0.025

However, we only found the linear relationship between a few face key points and two dimensions of the Big Five (Agreeableness and Openness.), this result suggests that faces are related to personality. We consider that the relationship between the face key points and personality traits is not only linear but maybe non-linear. Like the experiment (19), use all the face key points trained by the CNN model, and obtain a good result. Therefore, we attempted to train models with all the face key points later.

### Model building

In the previous correlation analysis, we found that the dimensions of Agreeableness and Openness are significantly correlated with facial movements (mainly linear correlations). However, there may be non-linear correlations with facial movements in the other three dimensions. Therefore, we attempted to find their non-linear correlation through computational learning algorithm models. Simultaneously, we used a linear model as a reference to compare it with other non-linear models.

### Feature extraction

We passed the data of 750 frames of each point to the *x*-axis and *y*-axis to the “tsfresh” (23) tool to obtain 30-time series features, such as minimum and maximum. All of the time series features are shown in Table 4. Each sample includes 4200 features [70 key points ^*^ two coordinates (*x* and *y*) ^*^ 30-time series features] through feature extraction.

**Table 4:** 30 time series features.

**Feature name**	**Description**
Maximum	Calculates the highest value in a set of values
Minimum	Calculates the lowest value in a set of values
Mean	Average of a set of values
Variance	Variance of a set of values
standard_deviation	Standard deviation of a set of values
Skewness	Sample skewness of a set of values (calculated with the adjusted Fisher-Pearson standardized moment coefficient G1)
Kurtosis	The kurtosis of a set of values (calculated with the adjusted Fisher-Pearson standardized moment coefficient G2)
Median	Median of a set of values
abs_energy	Absolute energy of a set of values which is the sum over the squared values
absolute_sum_of_changes	Sum over the absolute value of consecutive changes in a set of values
variance_larger_than_std	Denoting if the variance of a set of values is greater than its standard deviation. Return Int value
count_above_mean	Number of values in a set of values that are higher than the mean of itself
count_below_mean	Number of values in a set of values that are lower than the mean of itself
first_location_of_maximum	First location of the maximum value of a set of values
first_location_of_minimum	First location of the minimum value of a set of values
last_location_of_maximum	Relative last location of the maximum value of a set of values
last_location_of_minimum	Last location of the minimum value of a set of values
has_duplicate	Checks if any value in a set of values occurs more than once
has_duplicate_max	Checks if the maximum value of a set of values is observed more than once
has_duplicate_min	Checks if the minimum value of a set of values is observed more than once
longest_strike_above_mean	Length of the longest consecutive subsequence in a set of values that is bigger than the mean of itself
longest_strike_below_mean	Length of the longest consecutive subsequence in a set of values that is smaller than the mean of itself
mean_abs_change	Mean over the absolute differences between a set of values
mean_change	Mean over the absolute differences between a set of values
percentage_of_reoccurring_datapoints_to_all_datapoints	Percentage of unique values that are present in a set of values more than once
ratio_value_number_to_time_series_length	The factor is one if all values in a set of values occur only once and below one if this is not the case
sum_of_reoccurring_data_points	Sum of all data points that are present in a set of values more than once
sum_of_reoccurring_values	Sum of all values that are present in a set of values more than once
sum_values	Sum over a set of values
range	Calculates the range value of a set of values

### Feature dimensionality reduction

We have only 82 samples. Each sample included 4,200 feature dimensions. Compared to the number of samples, there are too many feature dimensions. In machine learning, too many features result in an overly complex model design. The complex model often leads to overfitting in the model training. Overfitting means that the model prediction accuracy on the training set is very high, but on the test set is low. Simultaneously, a complex model also renders the time and resource consuming during the model training process. Therefore, we used PCA to reduce the feature dimension. Due to the requirement of PCA feature dimensionality reduction, the number of feature dimensions after dimensionality reduction cannot be higher than the number of samples (82) and the total number of features (4,200). Consequently, we chose 25 and 30 dimensions as the number of feature dimensions after the principal components analysis (PCA) (24) dimensionality reduction for comparison. It's also consistent with the research results that the model training results are better when the number of PCA is ≥25 (25).

## Results

This study selected five commonly used regression machine learning algorithms, including LinearRegression (LR), SVR, RandomForestRegressor (RFR), XGBRegressor (XGB), and CatBoostRegressor (CBR), to construct an automatic identification model of the BFI-44 based on facial analysis. In the model evaluation, we used the three-fold cross-validation method and calculated the Pearson correlation coefficient between the predicted value of the training model and the value obtained by the BFI-44 to evaluate the model prediction accuracy. The results are shown in Table 5. It indicates that the prediction effect of CatBoostRegressor is best in the aggregate case, and the correlation coefficients of the five dimensions of BFI-44 all reach above 0.3 and about medium correlation.

**Table 5:** Results of the personality identification model.

**Model**	**A**		**C**		**E**		**N**		**O**	
	**Pearson**	**MAE**	**Pearson**	**MAE**	**Pearson**	**MAE**	**Pearson**	**MAE**	**Pearson**	**MAE**
**LR**										
PCA = 25	0.107	35.94	0.044	3.86	0.033	4.64	0.004	23.2	0.159	3.71
PCA = 30	0.15	34.5	0.177	3.77	−0.011	3.95	0.035	25.3	−0.009	4.48
**SVR**										
PCA = 25	0.231	3.59	0.174	3.42	0.043	3.59	−0.009	3.81	0.139	4.35
PCA = 30	0.166	2.88	0.313	3.12	0.138	3.2	0.088	3.85	0.158	3.96
**RFR**										
PCA = 25	0.338	2.23	0.349	3.28	0.318	4.65	0.423	2.77	0.359	2.86
PCA = 30	0.338	3.73	0.32	3.43	0.36	3.96	0.307	4.35	0.404	2.41
**XGB**										
PCA = 25	0.265	4	0.32	7.96	0.476	2.95	0.295	1.32	0.227	5.17
PCA = 30	0.341	8.69	0.388	6.94	0.307	2	0.293	2.67	0.382	1.87
**CBR**										
PCA = 25	**0.416**	4.4	**0.37**	4.66	**0.412**	3.14	**0.421**	4.39	**0.39**	3.33
PCA = 30	**0.395**	4.51	**0.385**	4.71	**0.387**	3.68	**0.386**	2.58	**0.432**	3.69

Bold values indicates the best model.

### Split-half reliability of the model

Reliability is a crucial evaluation index for the reliability of the training model, among which the Split-Half reliability is a conventional method for evaluating internal consistency. This experiment uses the odd-even frame split-half reliability to check the model reliability. We divided each frame of the original facial key point data into odd-numbered and even-numbered frame data. Subsequently, we used these two datasets to train two independent models and calculated the correlation coefficient between the scores of the above two models for personality prediction to evaluate the Split-Half reliability of the model in the five dimensions of the BFI-44 under the conditions of PCA = 25 and PCA = 30. The results are shown in Table 6. The results highlight that the Split-Half reliability of the model is better than 0.75 in different dimensions of the Big Five personality. It indicates that the trained model is reliable.

**Table 6:** Split-half reliability.

**Model**	**A**	**C**	**E**	**N**	**O**
CBR (PCA = 25)	0.96	0.927	0.756	0.815	0.864
CBR (PCA = 30)	0.931	0.96	0.824	0.834	0.883

## Discussion

The results (Table 2) of this study indicated that only the Agreeableness dimension showed a significant negative correlation with the facial point movement (*p* < 0.05). Furthermore, the relevant facial points were concentrated in the right jawline to the chin contour. Previous Studies support these results, and the study (26) shows that the accuracy of the right face is significantly higher than that of the left face in the emotional stability, intelligence/imagination, and health. Another study (27) indicates that emotionally stable, healthy individuals also showed higher scores in Agreeableness. It may be because the peoples with high Agreeableness scores have relatively stable emotions and relatively high mental health, and they do not readily show plenty of expressions on their faces, so there is a negative correlation between Agreeableness and facial movements. Furthermore, the higher the Agreeableness score, the smaller the facial movement amplitude.

From the *T*-test results in Table 3, we found a significant difference in the facial point changes on the left cheek's outer contour between the high and low groups on the Openness dimension (*p* < 0.05). The change range of the key points of the face is higher than that of the low Openness group. In previous studies, we found an evidence that people with more asymmetrical faces scored higher on Openness in one study (28). In addition, this explains why the higher the Openness score, the greater the facial movement, and why the difference appears only on one side of the face. However, it does not explain why the difference appears on the left cheek's outer contour. Consequently, we hypothesize that individuals with high scores in the Openness dimension have abundant expressions on the left cheek's outer contour than those with low scores and that the left cheek's outer contour has a greater range of motion.

In this study, we used five machine learning models for training based on the scores of the five dimensions of the Big Five. Subsequently, we calculated the Pearson correlation coefficient between the predicted value obtained by the training model and the value obtained by the subjects' BFI-44. The comparison indicates a certain degree of correlation between the predicted value and the value of BFI-44. However, there are also differences in the identification performance of each model. The results show that LR has the worst effect, indicating it's no simple linear relationship between the movement laws of various points on the face and the Big Five. RandomForest and CatBoost algorithm models, whether PCA = 25 or PCA = 30, have correlation coefficients above 0.3 in the five dimensions of personality. In addition, different algorithms show consistency higher than the correlation coefficient of 0.3, showing that it's feasible to identify the Big Five personality by face, and the CatBoost algorithm is best (29) in the aggregate case. The highest scores of the 5 dimensions appear in Agreeableness: CBR (PCA = 25, *R* = 0.416), Conscientiousness: XGB (PCA = 30, *R* = 0.388), Extraversion: XGB (PCA = 25, *R* = 0.476), Neuroticism: RFR (PCA = 25, *R* = 0.423), and Openness: CBR (PCA = 30, *R* = 0.432). Furthermore, different machine learning models have corresponding advantages for different dimensions of the Big Five personality, and differences in PCA will also affect the training effect of the algorithm. This result provides a direction for our follow-up research. We can explore more machine learning algorithm models and more PCA options. In addition, according to the result of the Split-Half reliability test, The value of Split-Half reliability on CBR in the case of PCA = 25 is 0.756, below 0.8, and other dimensions are higher than 0.8. Furthermore, even the Split-Half reliability values of individual dimensions are >0.95, which shows the high reliability of this experiment and provides reliable data support for in-depth research.

Although PCA is a good dimensionality reduction algorithm, the selected features cannot be backtracked after dimensionality reduction. This study abandons the unsupervised machine learning method (such as CNN) and uses the supervised learning method to find key points of the face that are closely related to the Big Five personality. After using the PCA dimensionality reduction algorithm to prove that supervised learning can also effectively identify the Big Five through facial video, follow-up experiments can use dimensionality reduction algorithms that can retain the original feature information to look for correlations between facial key points and the Big Five.

In addition, we used 750 frames of video data from each user as the foundation data, consulted related literature, and found no evident standards. We considered that more frame data contain rich information about personal movement and help personality identification. Simultaneously, excessive frame data affect the training efficiency and generalization of models. After comprehensively considering, we chose 30 s (750 frames) of video to train models. In further research, we should explore the influence of video duration on the accuracy of personality identification.

The number of samples recruited in this study was 88, and only 82 were left for use after denoising samples in the later stage. Too small learning samples directly affect the model generalization ability and prediction effect (30). In addition, the distribution of samples is relatively concentrated because 40% of the subjects were recruited from campus. Furthermore, the homogeneity of samples is serious. It also affects the model generalization ability and prediction effect. Future research should increase the sample size while covering various industries and age groups.

In this study, we recorded the subject videos at a fixed position and a fixed distance from the camera. Significant movement of the subject's head and the distance from the camera directly affect the value of the subject's facial point movement difference frame. If this study applies to actual usage scenarios, a person cannot stand in a fixed position to identify. Therefore, how to accurately identify the subject's facial features according to the camera distance in the dynamic is a higher requirement for our subsequent research.

## Conclusion

According to Pearson's correlation analysis, the high-low group *T*-test, and the model prediction results, this study proposes a new, feasible, and effective method for identifying personality traits through ordinary video analysis. This method solves a series of shortcomings of only using questionnaires (Learning effect, intentional concealment by the subjects, large-scale time-consuming, laborious, et al.), which presents the possibility for large-scale application. In addition, this study has limitations such as too few test samples, insufficient generalization of test samples, limitations of video shooting methods, and limitations of video identification capabilities. These limitations make the training of the model not optimal. Future research should seek to solve these problems in order to better apply to the realistic environment.

## Data availability statement

The datasets generated for this article are not readily available because the raw data cannot be made public. If necessary, we can provide feature data. Requests to access the datasets should be directed to the corresponding author.

## Ethics statement

The studies involving human participants were reviewed and approved by the scientific re-search ethics committee of the Chinese Academy of Sciences Institute of Psychology (H15010). The patients/participants provided their written informed consent to participate in this study. Written informed consent was obtained from the individual(s) for the publication of any potentially identifiable images or data included in this article.

## Author contributions

XL proposed the conception of this study and collected the essential data for this study. XL and LC jointly designed the experimental flow of this study. LC developed the tools needed for the experiment, executed the whole experiment process, performed all of the statistical analyses, and wrote the manuscript with input from all authors. All authors contributed to the article and approved the submitted version.

## Funding

This research was funded by the Key Research Program of the Chinese Academy of Sciences (No. ZDRW-XH-2019-4) and the Scientific Foundation of Institute of Psychology, Chinese Academy of Sciences (No. E2CX4735YZ).

## Conflict of interest

The authors declare that the research was conducted in the absence of any commercial or financial relationships that could be construed as a potential conflict of interest.

## Publisher's note

All claims expressed in this article are solely those of the authors and do not necessarily represent those of their affiliated organizations, or those of the publisher, the editors and the reviewers. Any product that may be evaluated in this article, or claim that may be made by its manufacturer, is not guaranteed or endorsed by the publisher.

## References

[1] Abe JAA Izard CE. A longitudinal study of emotion expression and personality relations in early development. J Pers Soc Psychol. (1999) 77:566. 10.1037/0022-3514.77.3.566 10510509

[2] Favaretto RM Araujo V Musse SR Vilanova F Costa AB. A software to detect OCC emotion, big-five personality and hofstede cultural dimensions of pedestrians from video sequences. arXiv. (2019) preprint arXiv:1908.06484. 10.1007/978-3-030-22078-5_8

[3] Anderson KW. Personality Factors Associated with Negative Affect: Application of the “Big Five” Taxonomy to Depression and Anxiety. Logan, UT: All Graduate Theses and Dissertations (1994) 3346. 10.26076/be2f-085a

[4] Bartone PT Eid J Johnsen BH Laberg JC Snook SA. Big five personality factors, hardiness, and social judgment as predictors of leader performance. Leadersh Organ Dev J. (2009) 30:498–521. 10.1108/01437730910981908

[5] Mikołajczak-Degrauwe K Brengman M Wauters B Rossi G. Does personality affect compulsive buying? An application of the big five personality model. In: Rossi G, editors. Psychology - Selected Papers. Croatia: InTech (2012), p. 131–44. ISBN: 978-953-51-0587-9 10.5772/39106

[6] Lilford N Vigar-Ellis D Nel D. Big Five personality traits and financial salesperson performance: an application of Chernoff faces. J Financ Serv Mark. (2014) 19:146–54. 10.1057/fsm.2014.10

[7] Hart JW Stasson MF Mahoney JM Story P. The big five and achievement motivation: Exploring the relationship between personality and a two-factor model of motivation. Indiv Differ Res. (2007) 5:267–74. Available online at: https://www.researchgate.net/publication/274080888_The_big_five_and_achievement_motivation_Exploring_the_relationship_between_personality_and_a_two-factor_model_of_motivation

[8] Gerber AS Huber GA Doherty D Dowling CM. The big five personality traits in the political arena. Annu Rev Polit Sci. (2011) 14:265–87. 10.1146/annurev-polisci-051010-111659

[9] Jani D. Relating travel personality to big five factors of personality. Tourism. (2014) 62:347−59. Available online at: https://hrcak.srce.hr/clanak/194846

[10] Van Dijk SDM Hanssen D Naarding P Lucassen PLBJ Comijs H Voshaar RO. Big Five personality traits and medically unexplained symptoms in later life. Eur Psychiatry. (2016) 38:23–30. 10.1016/j.eurpsy.2016.05.002 27611331

[11] Tok S Morali S. Trait emotional intelligence, the big five personality dimensions and academic success in physical education teacher candidates. Soc Behav Pers. (2009) 37:921–31. 10.2224/sbp.2009.37.7.921

[12] De Bortoli D da Costa N Jr. Goulart M Campara J. Personality traits and investor profile analysis: a behavioral finance study. PloS ONE. (2019) 14, e0214062. 10.1371/journal.pone.0214062 30917175 PMC6436746

[13] Rahman AU Halim Z. Predicting the big five personality traits from hand-written text features through semi-supervised learning. Multimed Tools Appl. (2022) 81, 33671–33687. 10.1007/s11042-022-13114-5

[14] Al Moubayed N Vazquez-Alvarez Y McKay A Vinciarelli A. Face-based automatic personality perception. In: Proceedings of the 22nd ACM international conference on Multimedia, 3–7 Nov 2014, (2014). pp. 1153–56. 10.1145/2647868.2655014

[15] Xu J Tian W Lv G Liu S Fan Y. Prediction of the big five personality traits using static facial images of college students with different academic backgrounds. IEEE Access. (2021) 9:76822–32. 10.1109/ACCESS.2021.3076989

[16] Halim Z Zouq A. On identification of big-five personality traits through choice of images in a real-world setting. Multimed Tools Appl. (2021) 80:33377–408. 10.1007/s11042-021-11419-5

[17] Ramon Y Farrokhnia RA Matz SC Martens D. Explainable AI for psychological profiling from behavioral data: an application to big five personality predictions from financial transaction records. Information. (2021) 12:518. 10.3390/info12120518

[18] Utami NA Maharani W Atastina I. Personality classification of facebook users according to big five personality using SVM (support vector machine) method. Procedia Comput Sci. (2021) 179:177–84. 10.1016/j.procs.2020.12.023

[19] Suen HY Hung KE Lin CL. TensorFlow-based automatic personality recognition used in asynchronous video interviews. IEEE Access. (2019) 7:61018–23. 10.1109/ACCESS.2019.2902863

[20] Ventura C Masip D Lapedriza A. Interpreting cnn models for apparent personality trait regression. In: Proceedings of the IEEE conference on computer vision and pattern recognition workshops. Honolulu, HI (2017), p. 55–63. 10.1109/CVPRW.2017.217

[21] Yesu K Shandilya S Rekharaj N Ankit K Sairam PS. Big five personality traits inference from five facial shapes using CNN. In: 2021 IEEE 4th International Conference on Computing, Power and Communication Technologies (GUCON). Kuala Lumpur: IEEE (2021), p. 1–6. 10.1109/GUCON50781.2021.9573895

[22] Cao Z Simon T Wei SE Sheikh Y. Realtime multi-person 2d pose estimation using part affinity fields. In: Proceedings of the IEEE conference on computer vision and pattern recognition. Honolulu, HI (2017), p. 7291–9). 10.1109/CVPR.2017.143

[23] Christ M Braun N Neuffer J Kempa-Liehr AW. Time series feature extraction on basis of scalable hypothesis tests (tsfresh–a python package). Neurocomputing. (2018) 307:72–7. 10.1016/j.neucom.2018.03.067

[24] Hotelling H. Analysis of a complex of statistical variables into principal components. J Educ Psychol. (1933) 24:417–41. 10.1037/h007132519405797

[25] Singh KK Chiranjeevi S Sivalal K. Face features-based personality assessment. In: International Conference on Computer Graphics, Visualization, Computer Vision and Image Processing 2021, CGVCVIP 2021, Connected Smart Cities 2021, CSC 2021 and Big Data Analytics, Data Mining and Computational Intelligence 2021, BIGDACI 2021 - Held at the 15th Multi-Conference on Computer Science and Information Systems, MCCSIS 2021. (2021). ISBN: 978-989-8704-32-0

[26] Kramer RS Ward R. Different signals of personality and health from the two sides of the face. Perception. (2011) 40:549–62. 10.1068/p6856 21882719

[27] Booth-Kewley S Vickers RR. Associations between major domains of personality and health behavior. J Pers. (1994) 62:281–98. 10.1111/j.1467-6494.1994.tb00298.x 7965560

[28] Fink B Neave N Manning JT Grammer K. Facial symmetry and the ‘big-five’ personality factors. Pers Individ Dif. (2005) 39:523–9. 10.1016/j.paid.2005.02.002

[29] Ibrahim AA Ridwan RL Muhamme MM. Comparison of the CatBoost classifier with other machine learning methods. Int J Adv Comput Sci Appl. (2020) 11:738–48. 10.14569/IJACSA.2020.011119032825442

[30] Cui Z Gong G. The effect of machine learning regression algorithms and sample size on individualized behavioral prediction with functional connectivity features. Neuroimage. (2018) 178:622–37. 10.1016/j.neuroimage.2018.06.001 29870817

